# LiMOX—A Point Cloud Lidar Model Toolbox Based on NVIDIA OptiX Ray Tracing Engine

**DOI:** 10.3390/s24061846

**Published:** 2024-03-13

**Authors:** Relindis Rott, David J. Ritter, Stefan Ladstätter, Oliver Nikolić, Marcus E. Hennecke

**Affiliations:** 1 Virtual Vehicle Research GbmH, 8010 Graz, Austria; 2 Joanneum Research—Digital Twin Lab, 9020 Klagenfurt, Austria; 3 Infineon Technologies Austria AG, 8020 Graz, Austria

**Keywords:** lidar sensor model, ray tracing, virtual testing, modular software architecture, infrared material database

## Abstract

Virtual testing and validation are building blocks in the development of autonomous systems, in particular autonomous driving. Perception sensor models gained more attention to cover the entire tool chain of the sense–plan–act cycle, in a realistic test setup. In the literature or state-of-the-art software tools various kinds of lidar sensor models are available. We present a point cloud lidar sensor model, based on ray tracing, developed for a modular software architecture, which can be used stand-alone. The model is highly parametrizable and designed as a toolbox to simulate different kinds of lidar sensors. It is linked to an infrared material database to incorporate physical sensor effects introduced by the ray–surface interaction. The maximum detectable range depends on the material reflectivity, which can be covered with this approach. The angular dependence and maximum range for different Lambertian target materials are studied. Point clouds from a scene in an urban street environment are compared for different sensor parameters.

## 1. Introduction and Motivation

Simulating lidar sensors is of interest in many different domains. There are various applications of lidar sensors, from airborne lidar over mapping and robotics to autonomous systems and autonomous driving. In this paper, we present a lidar simulation model for pulsed lidar sensors of the automotive domain, where the distance to an object is measured by the time-of-flight (ToF) principle. For automotive applications, lidar sensors are still expensive compared to radar sensors. However, they offer high-resolution perception of the environment in close and medium ranges (a few centimeters up to more than 100 m in most cases). Current lidar sensors used in advanced and autonomous driving are mostly pulsed lidar sensors, operating in the near-infrared (IR) spectrum. Thus, their maximum range and power emitted are limited by eye-safety protection criteria.

For virtual testing of autonomous driving functions, different types of sensor models exist. Some of them are purely based on object lists, as input and output. There are data-based, probabilistic, phenomenological or physical models. A good overview together with a classification of model fidelities is provided by Schlager et al. [1]. According to this classification, our model is a high-fidelity lidar model, since it generates point cloud data from 3D geometry models based on the measurement principle of the lidar sensor.

The research questions addressed in the present work are whether it is possible to develop ray tracing point cloud sensor models based on a modular or stand-alone software architecture, decoupled from direct integration in environment simulations (monolithic architecture), and to model realistic ray–surface interactions with respect to material properties in the IR spectrum with this approach. For object-list-based sensor models, a modular software architecture and integration into simulation frameworks is feasible [2,3], since the interfaces exist to transport object data, e.g., [4], while point cloud models need access to the entire 3D geometry and attributes thereof, such as materials associated at the polygon level.

To generate point clouds, two different approaches exist. One option is based on graphic shaders, which use the depth buffer or z-buffer on the GPU. This buffer holds depth information of the rendered image, equal to the distance of an object or primitive, like a polygon, from the camera or sensor, for each pixel. The other option is to perform ray tracing, where the path of a ray is traced from a point of origin (monostatic lidar sensor) up to the first surface encountered (also referred to as ray casting), or, if the surface is transparent for the simulated wavelength, up to the first reflective surface.

Our **Li**dar sensor **M**odel in **O**pti**X**, short LiMOX, is built upon the Nvidia ray tracing engine OptiX [5], which provides the framework for ray tracing parallelized on Nvidia GPUs. While there are various different ray tracing engines, such as Vulkan, DirectX (for GPU) or Intel Embree (which is CPU-accelerated), we chose OptiX, providing a basis for our stand-alone application. It leverages software and hardware acceleration on Nvidia GPUs, not only for ray tracing, but also for creating bounding volume hierarchies and updating objects efficiently in the scene to create scenarios. Additionally, the material handling and polygon-level assignment of material properties is facilitated by the OptiX engine. Its interoperability with the CUDA language provides additional benefits for the parallelization and execution speed, which was a design requirement to realize fast data generation in co-simulation environments, where CPU offloading is a major benefit.

Our motivation for the development of the model is twofold: the modeling of lidar sensor effects and the simulation of lidar sensors for the development of autonomous driving algorithms. Virtual testing of autonomous driving functions is a prerequisite to develop and improve them in controlled environments. While they can be tested with ground-truth data, sensor models extend the testing capabilities and contribute to a more realistic representation of the system [6]. A physically realistic sensor model with point cloud data output is not used by driving functions directly, because an object detection algorithm is needed. However, when simulating the sensor principle, it can be a synthetic input for perception algorithms. Given a suitably configurable sensor model, it is possible to test and compare various perception algorithms on a particular type of sensor using the simulated data. Conversely, it is also possible to test and compare various sensor types on a particular perception algorithm. This, for example, allows the effects of resolution, scan pattern or other sensor effects on the overall performance to be studied, and can be used to verify the requirement specifications for a lidar sensor. For a known perception algorithm, the sensor detection properties can be optimized by choosing appropriate sensor parameters, thus essentially creating an optimal sensor to support a given perception algorithm in the most efficient way.

Moreover, LiMOX is a stand-alone lidar model, which uses 3D geometry models, an IR material database and messages of the Open Simulation Interface (OSI) [4] type based on Google Protocol Buffer [7], for the dynamic update as an input, as opposed to models which are directly integrated in an environment simulator. It is designed for a modular software architecture which is based on the co-simulation of different models (environment simulation, sensor model, driving function, etc.) [6]. This supports the strategy that sensor models are not only developed as part of, or as a plug-in (API-based) to, an environment simulator, but can be created by, e.g., the sensor manufacturer directly. Many details of the sensor operation principle are unknown to third parties due to intellectual property rights, which can be best modeled by the manufacturer.

The highlights of this work are:

- A stand-alone ray tracing lidar model for a modular simulation architecture;
- Material classes to handle reflection, transmission and absorption;
- Ray–surface interaction based on IR surface material properties and incidence angle;
- Novel reflectivity-driven range handling including an IR measurement database;
- Ray tracing time-profiling results.

**Figure 1:**
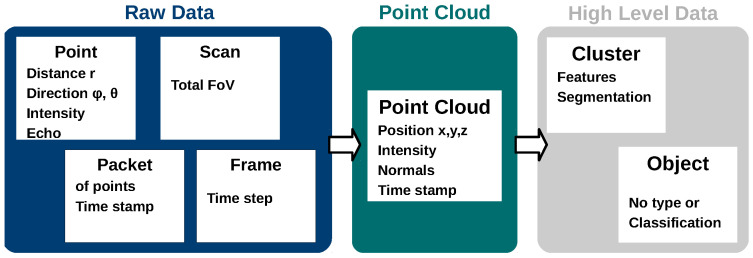
Overview of lidar data types processed in or outside the sensor. LiMOX focuses on point cloud data generation.

It is important to state that LiMOX is not a sensor system model and, therefore, does not cover the emitter and detector system, signal processing, post-processing or object detection. Instead, it is meant as a toolbox for a lidar sensor’s interaction with the environment and suited for the parametrization to a specific sensor’s ray pattern. Figure 1 provides a rough overview of possible types of lidar data which are processed in a sensor or during post-processing. However, not all the types listed typically occur in a single sensor. Our model focuses on the data type point cloud. The aim is to generate realistic point clouds, while the exact signal processing of, e.g., the return signal, single frames or scans as well as the temporal resolution of the shooting pattern down to the update frequency are neglected in the current version of the model. While lidar sensors operate on extremely high data rates (e.g., Ouster OS1 can detect 128 × 1024 points at 20 Hz), these are mostly not needed in simulation, since it is hard to process them within the time constraints given by a virtual simulation environment. This is due to the fact that the (co-)simulation environment needs to handle multiple different models. Handling high data rates can slow down the simulation significantly.

Another benefit of this model is the generation of point clouds in a fully controllable fashion. As opposed to models which are based on environment simulators, there is no hidden information; inputs, parametrization and execution are fully determinable. With our model, we study sensor effects and generate point clouds in a user-defined, controlled environment.

## 2. Previous Work

In the following, we focus on lidar models, which represent sensor types that can be applied to the automotive domain. Most of the models in the literature can be roughly associated to these categories:

(a) Developed for a simulator tool, either
  - Integrated/included in the simulator and parametrizable or
  - Programmable via an API or interface of the simulator.
(b) Modular:
  - Stand-alone or
  - Connectable
  with open interfaces.

There are many sensor models of different fidelity levels, which are part of environment simulation tools, such as VTD [8], CARLA [9] or CarMaker [10]. Either they are provided by the environment simulation and customizable by the user, or the environment simulation provides a programmable interface (API) to integrate third-party sensor model software directly.

An example of a third-party model integrated via an API is presented in [11]. The model is implemented as a sensor plug-in to the simulation framework VTD [8], based on the OptiX engine. Thus, the ray tracing is performed directly within the graphics pipeline with GPU acceleration. The main difference in our contribution is that ray tracing is performed stand-alone on 3D mesh models directly, using the OptiX engine for ray tracing and object handling, such as scene updating. Another difference is that [11] uses a camera model for ray tracing, exploiting different sampling grids for shooting rays into the scene, while in our model ray patterns are unconstrained. Similarly to our work, reflection models are included, material databases can be used and an intensity detection limit is applied, based on the lidar equation. One difference is that we do not consider detected intensities, but integrate material reflectivities directly.

Another example of a GPU-based lidar sensor model, which uses the raw-signal programmable interface of CarMaker for ray tracing, can be found in [12]. The model provides the open interfaces OSI and FMI for data exchange. Both raw data and object data can be generated.

In the following, we will limit this overview to sensor models which were created as separate software modules, since they reflect the modular software architecture of interest.

**Figure 2:**
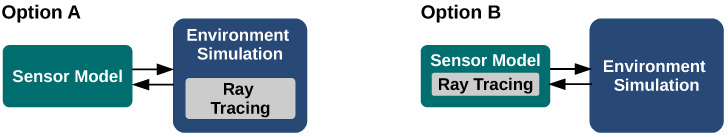
Different options for modular sensor models based on ray tracing. Generally, ray tracing is performed directly in the environment simulation tool (option A). In LiMOX, ray tracing is performed within the sensor model (option B). Due to the internal handling and update of objects, it can even be operated stand-alone.

A best-practice example of a modular high-fidelity lidar sensor model is presented in [13]. The model represents a specific lidar sensor by Blickfeld, and includes the entire signal processing tool chain. It uses the standardized interfaces FMI and OSI for co-simulation. A realistic lidar point cloud is generated based on the scan pattern and the interaction with a 3D simulation tool. To demonstrate the modular applicability, two simulator tools are used to generate results. Finally, the resulting point clouds are compared to measurement data from the modeled sensor. Compared to their specific virtual sensor model, our toolbox does not aim to provide a digital twin of a specific sensor on the market. A particular difference is that ray tracing in LiMOX is performed stand-alone and independently of any simulator tool; see Figure 2.

The advantage of graphics-shader-based models is that the information needed is available in the graphics pipeline and the shader model can be easily integrated into the pipeline. There is no need for special interfaces or the generation of a digital twin of the simulation environment. A lidar model which can be included directly in the rendering pipeline is GLIDAR [14]. It is based on OpenGL [15] with a specific fragment shader retrieving data from the depth buffer. The accuracy of the depth buffer is increased by the model through usage of different color channels, and depth banding (degradation of depth accuracy with distance to the view point) is avoided.

A ray tracer needs access to the full set of geometry models in the scene together with other information on the 3D surface properties. These interfaces and exchange formats for 3D models are not standardized. There are many different formats to define geometry models and they cannot be accessed easily in a simulation environment during runtime. Therefore, it is often necessary to create a digital twin for the sensor model. Some lidar models in the literature solved this problem by choosing a graphics software tool to build the model upon. The VTK lidar simulator presented in [16] uses the Visualization Tool Kit (VTK) [17], a versatile, open-source graphics software toolkit. The lidar scanner performs an intersection test of a ray with the 3D mesh models to generate a point cloud. The simulator can also create a 3D mesh model from a given point cloud via Delaunay triangulation. The software is an extension of the object-oriented API in VTK and introduces new classes for rays and hit points. A noise model is included to create realistic sensor data. BlenSor [18] is another model, based on an open-source graphics software, Blender [19]. BlenSor’s lidar simulation toolbox performs ray casting in Python. The reflection properties of the surface hit by the ray are considered with respect to the surface material of the geometry model. The reflection model used is provided by Blender. Line scanners, mechanically rotating lidar sensors as well as ToF cameras can be simulated.

HELIOS++ [20] is a simulation environment which exceeds the lidar simulation and provides a full survey, including different sensor platforms and environments. Ground-based and airborne platforms can be chosen. Besides static scenes, the model provides continuous measurement data from the moving platform, based on a trajectory provided. A very high control over the geometry inputs and realistic trajectories of moving sensor platforms are supported. Beam divergence, via subsampling and full-waveform simulation, is covered and different scanning patterns and detector principles are predefined.

A sequential lidar system simulator is presented by Rosenberger et al. [21]. The simulation is based on a modular approach using co-simulation, and can operate on both object data or point cloud data. Using the sensor output of existing free or commercial environment simulators, a modular sensor framework is used for post-processing raw point clouds or object data up to different sensor interface levels, from realistic point clouds to segmentation, tracking and object classification. The framework uses OSI messages and the modules are Functional Mock-up Units (FMUs) [22] for model packaging.

Lidar system simulation based on parallel ray tracing including a comparison with measurement data is presented by Gusmao et al. [23]. The simulation is based on NVIDIA’s OptiX ray tracing engine. Point clouds are generated for mesh models individually and later combined to a synthetic point cloud. A programming library is used to remove redundant points. Surface normals and color are included in the synthetic point cloud data. As opposed to their approach, we use a sensor origin location from which the rays are launched to simulate a lidar sensor. This mimics the physical shooting principle of the sensor, without generating any redundant points.

A lidar emulator developed for terrestrial lidar detection for static or moving platforms is presented by the authors of [24], operating on 3D synthetic scenes. It is based on OpenGL and performs GPU-accelerated ray tracing, providing spatial, semantic and intensity outputs. Similarly to our work, it considers material properties. Their model differs in the way it implements the ray surface intersection algorithms directly, and rays are constructed iteratively. In our model, rays are created in parallel by the inter-operation of OptiX with CUDA buffers, where they are stored. While they use different materials and BRDFs for the reflection and intensity approximation, there are no details provided on the underlying IR material models (BRDFs) or the material database used.

Another stand-alone lidar simulator is presented in [25], where the authors tackle the problem of realistic 3D mesh models as inputs to ray tracing models. For this, they develop a database of 3D models, based on real world measurement data, apply ray casting onto these models and finally use deep neural networks to introduce realistic properties to their sensor model. Thus, their model is highly data-driven, and differs from our approach, simulating the physical interaction of the lidar sensor with the object surfaces detected. However, in our approach, we are not limited to 3D mesh models from environment simulators, but can also use more realistic 3D meshes, e.g., from high-resolution lidar object-reconstruction tool chains.

Building upon a similar idea, lidar point cloud generation is performed in PCGen [26]. The simulation pipeline uses a combination of simulated and real-world data and enhances the data via deep learning. The authors focus on the point cloud generation of vulnerable road users to train object detection algorithms.

Our approach differs from the aforementioned models. Our contribution in this work is a modular lidar sensor model, based on a fully controllable stand-alone application, with a novel approach to handle different materials. We introduce material models based on surface reflectivity measurements and material classes to consider reflection, transmission and absorption properties. The material reflection data are loaded from an IR material database. Maximum range–reflectivity relations impact the generated point cloud directly.

Ray tracing is performed entirely within the model; see Figure 2 option B. Thus, it is fully controllable and can be applied independently of the environment simulation tool. For efficient execution and real-time capabilities, a GPU acceleration for ray tracing and accelerated scene updating is chosen, which allows CPU offloading, e.g., for co-simulation applications. The advantage of a modular sensor model is the combination with various other simulation tools, as is expected of sensor models from sensor manufacturers, for example.

Here is an overview of the paper’s structure. Our stand-alone lidar model uses NVIDIA’s OptiX engine to perform ray tracing from a sensor origin point on a 3D environment created by loading 3D geometry objects to defined positions in a scene, as shown in Section 3. Each polygon of the geometry model can be assigned a material from an IR material database, and ray–surface interactions based on the incidence angle and material reflectivity can be simulated, as shown in Section 4. The influence of the maximum detectable range with respect to the reflectivity is considered in the model. Different functional relations can be used for the range–reflectivity dependence (Section 4.2). Object positons and orientations can be updated dynamically to simulate a scenario. The applications and limits are discussed in Section 5. Abbreviations and the nomenclature are given in the Nomenclature section.

## 3. Model Description

The ray tracing principle, which our model is built upon, resembles the sensor principle of a lidar sensor, sending a ray of infrared light from a source in a particular direction in space. In the model, we simulate pulsed lidar sensors. For the hit point on a reflective surface, the reflectivity is stored together with the point’s location in the point cloud.

Since ray tracing is well suited for parallelization, the model is based on the ray tracing engine OptiX by NVIDIA, which can be run on NVIDIA GPUs. OptiX is a stand-alone graphics API, which simplifies the implementation and execution of ray tracing applications and provides hardware acceleration in the form of parallelization and hierarchical space decomposition. For virtual testing of autonomous driving scenarios, the sensor model was designed to be part of a modular software-in-the-loop architecture, based on co-simulation. In this application, the performance of each model limits the virtual testing speed. Therefore, OptiX is a good choice to increase model performance and to handle the necessary update frequency of the scenario. Moreover, OptiX is interoperable with NVIDIA’s CUDA programming interface, which provides a further extension to OptiX.

LiMOX can be used either to create point clouds for a given static scene, or an entire scenario. The dynamic update phase of the stand-alone lidar model together with time profiling is presented in [27].

The advantage of the stand-alone model is that the data can be generated completely independently of any environment simulation. Only 3D mesh models of the environment and the vehicles, together with OSI data including a scenario, are needed. The OSI data can be saved to compressed data files on the disk or published via a TCP/IP server. After the initialization, the sensor model reads the OSI data and updates the scene graph. The results are written to PCD files [28] and can be post-processed by software tools, e.g., the open-source tool pointcloudset in Python [29], or used directly by another application. Therefore, no additional software component is necessary to create point clouds.

To connect the model to an environment simulation, 3D geometry models from the simulator need to be accessible together with an update using OSI data. With this approach, there are a couple of unresolved issues in the interaction with the simulator. In particular, the mapping of IR material properties, the integration of mesh substructures, such as bump-maps or displacement maps, as well as animation effects are not covered by the interfaces used. Moreover, the stand-alone model includes a lot of overhead in object handling and updating the scene graph.

### 3.1. Model Inputs

At the object level, the inputs of the model are 3D geometry meshes with material association, access to an IR material database and a mapping of all mesh materials to appropriate IR database materials. Moreover, the object poses in the scene need to be provided. This is performed in the initialization files and through dynamic update using OSI messages.

**Figure 3:**
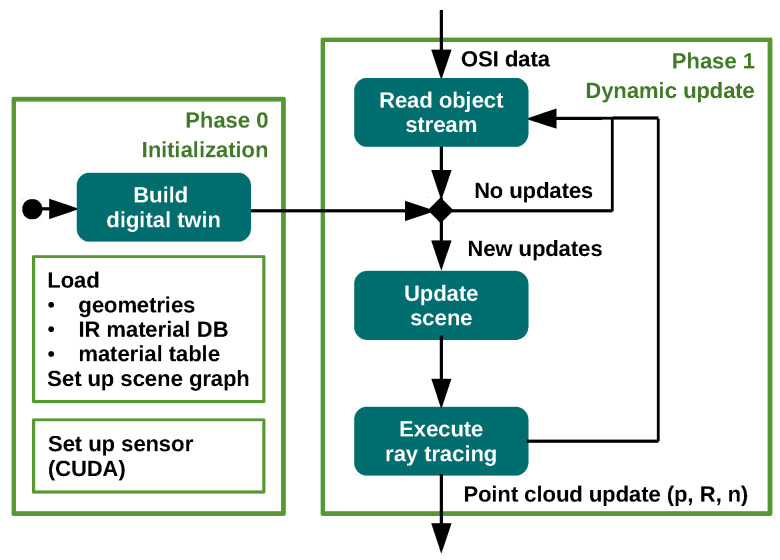
Overview of the model and its update procedure, see also [27].

There are two operation phases of the model, an initialization phase and the dynamic update phase, shown in Figure 3 and presented in [27]. In the initialization phase, the sensor parameters and the 3D scene need to be set up. First, the geometries and the IR material database are loaded. Next, the mapping of materials for each geometry mesh is performed. The materials of the 3D mesh models are typically defined for visual rendering purposes only and therefore need to be assigned to appropriate materials from the IR material database used. A representative mapping from materials defined for visual rendering to appropriate materials of the IR material databases is still an open research question, since the underlying physical material is often unknown. A mapping table of all mesh materials is provided to the model. Finally, the OptiX scene graph is established and the sensor is set up using CUDA buffers.

In the dynamic update phase, the location and the orientation together with a unique ID are provided for each geometry model or object. Dynamic objects can be updated via OSI using their unique IDs in the scenario. In each time step, the information is updated. New OSI data are compared to the previous state of each object. If any changes to the object states are detected, the objects are updated and transformed and a ray tracing execution is launched. Thus, point cloud data are not generated continuously as in a physical sensor, but only when there are changes in the scene, which is resource-efficient.

Some of the geometry meshes used for generating point clouds presented in this paper are provided by courtesy of the environment simulation VTD [8], by Hexagon. VTD includes a large database of geometry models together with an OSI message interface. Thus, it is a possible candidate to connect our lidar model to.

### 3.2. Model Outputs

In the created point clouds, three parameters are stored for each point: the point location p, the material reflectivity *R* and the face normal n of the hit polygon.

The point location is given in Cartesian coordinates. The reflectivity of each point is retrieved from the material hit by the lidar ray and the angle of the ray–surface interaction. Details on the material model and the reflectivity are presented in Section 4.1. Since a monostatic sensor (emitter and detector are at roughly the same location) is modeled, only the reflectivity with respect to the angle of the incident ray is of interest. The value is retrieved from the IR database loaded in the simulation.

### 3.3. Parametrization

The model is used as a toolbox to simulate a broad range of different sensors for automotive applications. Therefore, the focus is not on the simulation of the full sensor system for a specific sensor or manufacturer model. Operation details like time resolution of the shooting or detection pattern, simulating the update frequency or post-processing-related issues such as dynamic range attenuation are currently not covered. Instead, the maximum number of possible ray directions is generated in one time step and the number of point clouds generated is not related to the sensor’s update frequency.

A parametrization of the model with respect to sensor parameters such as ray pattern, field of view and maximum range is possible. For the parametrization, sensor origin and ray directions are defined. A minimum and maximum range of the sensor can be defined, independently of the material properties. Additionally, range–reflectivity value pairs can be defined.

There are several options to define ray directions. Eight predefined sensors (Velodyne sensor VLP-16, Puck HiRes, HDL-32 and HDL-64, Ouster sensor OS1 with 16, 64 and 128 layers and Ibeo 4L) can be chosen directly using a name tag. The second option is to define the field of view in the vertical and horizontal directions together with the respective resolutions. The third option is to provide an array of vertical and an array of horizontal ray angles, which are combined to generate a regular grid pattern. Alternatively, an array of angle pairs for irregular patterns (no grid structure) can be provided to define the ray directions.

**Figure 4:**
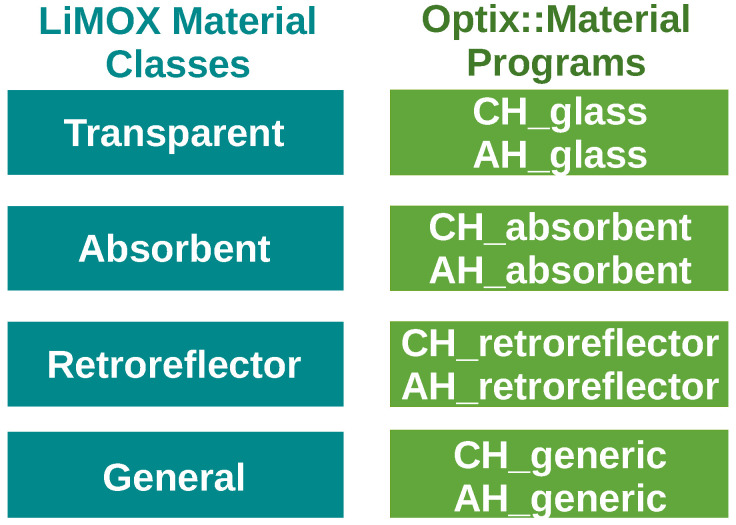
Material classes with different hit programs in OptiX, for closest hit (CH) and any hit (AH), as defined by the OptiX engine’s SDK.

Sensor effects can highly differ for different sensors considered. In OptiX, different material programs can be defined to handle different types or classes of materials. These material hit programs can be used to generate more specific models, which reflect the sensor effects observed in different sensor types or from measurement data, such as the signal processing of retroreflective targets. The different material hit programs and classes we use in the model are presented in Figure 4. In addition, using range–reflectivity parameters from a sensor’s data sheet allows us to model material-dependent ranges.

In general, parameters for the lidar sensor model can be retrieved from three different sources. In Table 1, the parameters used in the model together with their sources are shown. Apart from parameters from data sheets, another source of parameters is data analysis of measurement data. Sensor effects like the influence of weather conditions need to be deduced from data analysis and stored in a database or lookup table. Moreover, the lidar sensor model needs a number of parameters which are directly from the setup of the simulated scene or scenario, such as material parameters or the input of the current external weather condition.

## 4. Sensor Effects

An important objective for LiMOX is to model sensor effects. The effects on the sensor can be roughly separated into two groups: effects caused by, or inherent to, the lidar principle and interactions with the environment (intrinsic), and effects caused by the operation principle of a specific lidar sensor due to its internal operation and processing (extrinsic). Extrinsic sensor effects depend on the specific sensor used. To model them in simulation, prior knowledge of the particular sensor and its internal operation is needed. Modeling these effects for one lidar sensor does not guarantee that the effect is applicable to models of other lidar sensors.

Since our model does not cover the full system simulation but focuses on the interaction with the environment and is parametrizable for different sensor types, we are mainly interested in integrating the intrinsic sensor effects.

In the following, we present the handling of material surface properties in the model and the effects that can be covered with this approach.

### 4.1. Material Model

Each point of the point cloud not only holds the coordinate, but also the reflectivity of the point hit by the lidar ray. Since the beam divergence is currently not modeled, there is no direct relation to the area hit.

Thus, a material model that describes the ray–surface interaction is needed. For this, special material classes are introduced to handle material attributes. We neglect the transmission or absorption parts of the lidar ray for the general material class. These material attributes are handled by the material classes defined for absorbent and transmissive materials. For a reflective surface which can be detected by the lidar sensor, only the reflective part needs to be considered. The reflectivity *R* of a surface is the ratio of reflected power to incident power at the first surface encountered, typically given in percent. For homogeneous and semi-infinite media, the material reflectivity is equivalent to the reflectance ρ of the material, R=ρ, where the reflectance describes the ability of a material to reflect radiant energy, also in percent. It mostly differs for thin-layered materials with transparent attributes causing multi-reflections, like window glass, which are not considered at this point. In a first approach, we only handle fully transparent and non-transparent surfaces.

Material reflectance is typically modeled using a bidirectional reflectance distribution function (BRDF). While there are various possible BRDFs, further simplifications can be assumed for lidar sensors. The ray is emitted from a point light source. For the lidar detector of a monostatic sensor, only one direction is of interest, which is the same for the incident and reflection angles of the surface hit. Therefore, instead of a BRDF, database measurements are used. LiMOX can use material reflectance data $\rho_{\lambda }$ from two different sources:

- NASA ECOSTRESS library [30] (assuming a Lambertian target);
- IR-measurement material database (IR database) [31,32,33] (angle dependence for 10° bins).

The NASA ECOSTRESS library [30] consists of a collection of around 3400 spectra of natural and man-made materials, covering the visible and thermal IR spectra (λ∈[0.35,13]μm). Thus, the exact wavelength of the lidar sensor simulated can be chosen; however, the reflectance corresponds to the incidence angle of θ=0°. The angular dependence of values used from the ECOSTRESS spectral library are retrieved with the assumption of a Lambertian target (BRDF), which represents an ideal diffuse material. The reflectance $\rho_{\lambda }\left(\theta \right)$ is given by Lambert’s cosine law

(1) $$\rho_{\lambda }\left(\theta \right)=\rho_{\lambda }\left(0\right)\cdot cos\left(\theta \right)\;.$$

The IR database [31,32,33] was created by Ritter et al. and is being continuously extended [34]. It currently holds over 200 different material measurements and is publicly available [32,33]. The measurements were performed using a ToF camera as light source operating at λ=945 nm and the measurement setup was designed to collect angle measurements of a surface in 10° steps of the incidence angle. Thus, there are nine bins of angle measurements taken at discrete angles θ∈[0°,80°] covering 0° to 90° incidence angle. The reflectance values are given in percent with respect to a Lambertian target at 0° incidence angle. The advantage of this database is that no BRDF model needs to be assumed. However, the wavelength is fixed in simulation to 945 nm. Most lidar emitters use sources from 840 nm up to 1050 nm wavelength. For many materials, the spectrum does not change significantly over this range, as shown in [31].

#### 4.1.1. Material Classes

For the ray–surface interaction, any material is associated with one of four different material classes:

- Transparent;
- Absorbent;
- Retroreflective;
- General materials.

This classification enables the definition of separate methods, or hit programs in OptiX, as shown in Figure 4, for different material types and collected materials which show similar properties in the IR spectrum. Most materials fall into the general material class defined, which yields the reflectivity of the material with respect to the incident angle of the ray. Some materials are transparent in the IR spectrum, or at certain incident angles other materials absorb infrared light. For transparent materials, the hit program of this class will ignore the surface encountered. For absorbent materials, the ray is canceled at that surface.

A known material can be assigned to a material class depending on its reflective property. An example is frosted glass, which does not need to be assigned to the transparent material class, since it has a high reflectivity. It can easily be added to the general material class and is treated as a reflective surface with negligible transmission. For materials which show different properties under different angles, e.g., with angles of total reflection, new material classes can be easily introduced.

Retroreflective materials reflect the incident light back into the incident direction only. Thus, the sensor can become saturated, or the detected intensity is significantly higher than the intensity of other types of materials (dynamic range effect). In the sensor, high intensities are often processed differently, which could be accounted for in the model as well. An adaptation of the LiMOX model with respect to a known sensor becomes possible.

The sensor model can be extended based on measurement data of a specific lidar sensor to further improve or extend the OptiX hit programs or the material classes used. If there are additional material classes needed, they can easily be added to the model to cover more phenomena.

#### 4.1.2. Material Mapping

**Figure 5:**
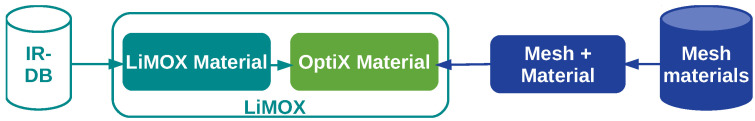
Material mapping using IR material database and 3D mesh models together with LiMOX material classes and OptiX material programs.

A major challenge is to assign each material from the visual material database, used in 3D mesh models or by environment simulations, with an IR material which corresponds to the surface modeled by the simulation environment. In Figure 5, the access of the model to IR and visual materials, via databases or geometry meshes, is shown. The IR database is handled by the LiMOX material classes and their corresponding OptiX materials with the defined hit programs, for closest (CH) or any hit (AH), depicted in Figure 4.

For the task of assigning each visual material a representative IR counterpart, meta information is needed. The appearance of a visual material can be similar for different physical materials, while the underlying physical properties differ a lot in the IR spectrum.

Visual materials are represented by reflection models, e.g., the Phong model, and typically consist of ambient, diffuse and specular reflection properties. Another type of materials used in computer graphics are physically based rendering (PBR) materials. PBR materials provide a better description of the physical properties like surface roughness and metal property. Still, there is no reference to the underlying material that the surface is composed of.

Since it is not possible to define a general map for visual and IR materials, a lookup table needs to be defined individually for the geometry meshes and the visual materials they contain or the visual material database used. This lookup table is loaded by LiMOX to map the visual materials defined for the meshes to the associated IR materials.

**Figure 6:**
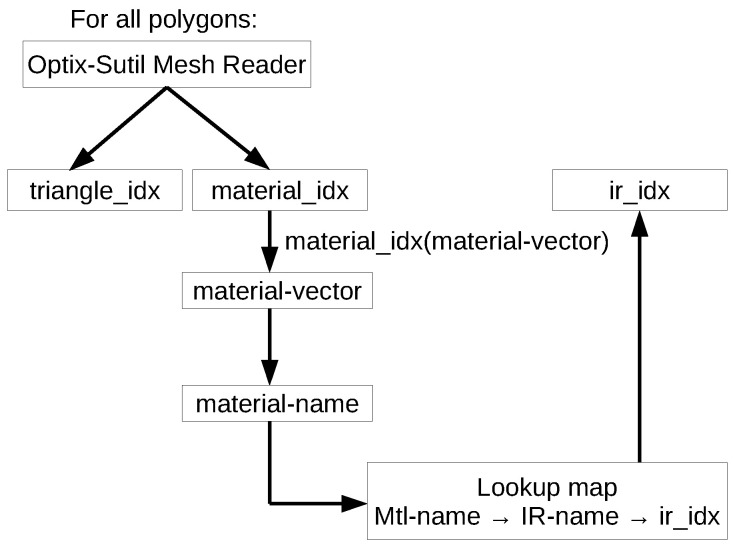
Mapping at polygon level of visual materials defined in the geometry mesh to materials of the IR database.

In Figure 6, the procedure for mapping the material associated with each polygon is sketched. An OptiX utility is used to load the meshes of the Wavefront OBJ format [35]. The materials are defined, e.g., in Wavefront Material Template Library (mtl) files [36]. Each polygon of the geometry mesh holds a material index. This material index relates to a vector of the materials defined within the model, which have a name tag. The lookup table is used to map each visual material to its IR counterpart. The IR database is loaded and an index is assigned to each IR material, which can be assigned as the corresponding IR index for each polygon, according to the mapping table. This IR index is used in the OptiX intersection program to call the appropriate hit program defined for the OptiX material class the material belongs to and possibly calculate the reflectivity from the material entry of the IR database.

### 4.2. Range–Reflectivity Dependence

The correct mapping of the mesh materials to the IR database is an open topic, which can best be solved by defining the material properties in the IR spectrum directly during mesh generation. With visual material properties only, it is hard to generate realistic reflectivity values for the scene. However, besides the problem of assigning each surface the correct IR material, the maximum range with respect to the material reflectivities can be handled separately and is used in the model for generating the point clouds.

Sensor data sheets typically include the maximum range of a lidar sensor with respect to the reflectivity of a Lambertian target. Ideally two value pairs are given, one the reflectivity at a low level (e.g., 10%) with its maximum detectable range and one the reflectivity at a high level (e.g., 80%) with its according maximum range. These two value pairs can be used to generate a curve fit and find a maximum distance limit for all reflectivities.

**Figure 7:**
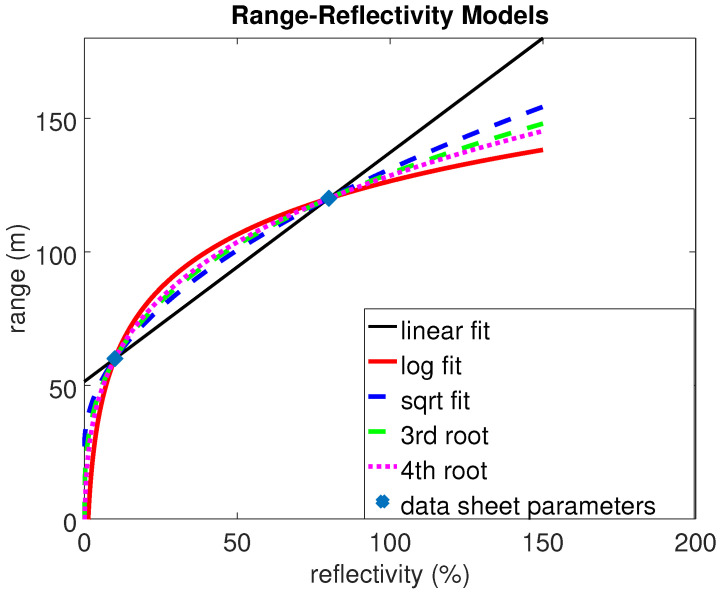
Different functions can be assumed for the range–reflectivity relation.

For each hit point, the range is checked and the point is only kept in the point cloud if the distance to the sensor does not exceed the limit for the reflectivity of the surface. Different curve fits are shown in Figure 7 to model the range–reflectivity dependence, together with the values provided in the data sheet. The most simple choice to extend the relation to all reflectivities is a linear fit; however, it is not physically motivated. The intensity of a light source typically decreases with $1/r^{2}$. The detected signal is also influenced by the intersection area of the lidar beam and the target, according to the lidar range equation [31,37]. The exponential factor varies with the extension of the target compared to the cross-section of the lidar beam. It ranges from $1/r^{2}$ for an area or extended target (target larger than beam, fully blocks the beam) over $1/r^{3}$ for a linear or wire target (beam fully covered in one dimension) to $1/r^{4}$ for a point target (target smaller than beam). Considering this, the following relation can be assumed between the range limit $r_{L}\left(R\right)$ and material reflectivity *R*:

(2) $$r_{L}\left(R\right)\propto R^{\frac{1}{n}}$$

where n∈[2,3,4]. In Figure 7, the linear fit is compared to these physically motivated fit functions of root two, three and four. Additionally, a logarithmic fit is shown for comparison.

The choice of an appropriate range limit curve has a large impact on the point clouds created. There are various effects, which need to be considered, such as the beam divergence or the dynamic range. While both effects are not directly modeled in LiMOX, they can be covered by an appropriate model for the range–reflectivity limits. At large distances (where only higher reflectivities can be detected), for example, the beam divergence becomes a predominant factor, while at short distances the dynamic range has a major impact on the detection and the maximum range of low reflectivities. These relations can be used to generate a user-defined range limit curve composed of different functional relations for a known sensor system, applicable in our toolbox model.

A further analysis and derivation of atmospheric conditions and weather effects and their impact on the range–reflectivity limits can be found in [38]. Another detailed analysis of weather effects from rain and fog and their respective simulation results are shown in [39].

**Figure 8:**
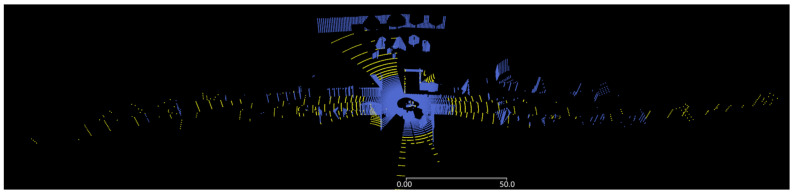
Top-side view of lidar point cloud when range–reflectivity limit curve is active (blue) or tuned off with a constant maximum range for any reflectivity (yellow and blue). All surfaces are modeled with Lambertian 95% target.

In Figure 8, the impact of a range–reflectivity limit curve is visualized. The point cloud of the Ouster sensor with 128 layers is shown for an active range–reflectivity limit given by two value pairs of [10%, 60 m] and [80%, 120 m] with a logarithmic fit in blue, and is compared to a constant maximum range limit for all reflectivities (1000 m) in yellow. All surfaces are covered with the Lambertian 95% target as the material.

**Figure 9:**
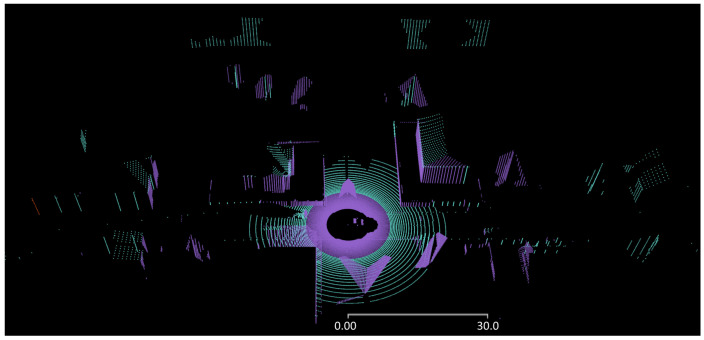
Comparison of range–reflectivity dependence for two point clouds using Lambertian targets of 10% reflectivity (purple) and 50% reflectivity (green) for all surfaces, as seen obliquely from above the street. Green point cloud contains the purple point cloud.

Using the same value pairs as before, with the logarithmic fit function, a comparison of two point clouds resulting when using different Lambertian targets of 10% and 50% for all surfaces, respectively, is shown in Figure 9. To use a single material from the database for all surfaces is certainly not physically correct. However, this allows us to decouple the impact of the material mapping from the angle dependence of the material reflectivity considered in the ray–surface interaction together with the range limits based on reflectivities. This is apparent in Figure 9 for all surfaces hit under large angles from the face normal, such as rooftops of buildings or the ground plane at larger distances from the sensor.

## 5. Applications and Limits

Our model can be used to generate lidar point cloud data from given a scene or scenario. The advantage of the stand-alone model is that the data can be generated completely independently from other software tools.

To generate a point cloud from a scene, 3D geometry meshes of the environment and the vehicles together with their poses and the sensor setup are the sole inputs. Pedestrians or bicycles are currently not covered. For these object classes, micro-motion, like arm or leg movement, makes up a non-negligible percentage of their motion. Thus, object animation is needed in these cases.

An entire scenario is provided to the model by OSI data, holding object updates with IDs. After the initialization, the established scene graph is updated by reading the OSI messages. Results can be written to PCD files [28] and post-processed or used directly by another application. In [27], the dynamic update procedure of a scene is presented and a use case is shown.

**Figure 10:**
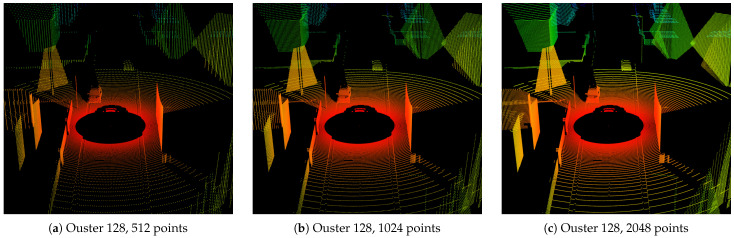
Comparison of different horizontal resolutions of the Ouster OS1 sensor with 128 vertical layers, (**a**) 512 points (0.7°), (**b**) 1024 points (0.35°) and (**c**) 2048 points (0.18°) over 360° in each layer. A supplementary animation of the scenario with sensor parameterization (**b**) can be found in the Supplementary Materials.

Besides creating raw point cloud data, it is possible to compare different sensor types and ray patterns for the same scene or scenario. A detailed comparison of different sensor parameters can be studied. This includes the variation of a single sensor parameter, e.g., the horizontal resolution, which directly impacts object detection and classification. In Figure 10, the point clouds of the Ouster sensor OS1 with 128 layers are presented for different horizontal resolutions.

### 5.1. Performance

Due to the parallelization on the GPU, the timings of ray tracing alone scale well for doubling the number of rays. Without any additional optimizations, the ray tracing times of the scene shown in Figure 10 are (a) ∼1 ms (128 × 512 rays), (b) ∼1.16 ms (128 × 1024 rays) and (c) ∼1.35 ms (128 × 2048 rays), which equals an increase of ∼16% for doubling the number of rays in each step, see also Table 2. This is only a rough comparison, performed on a NVIDIA RTX 2080 GPU, with RT cores inactive, since the absolute times highly depend on the hardware used.

In general, ray tracing times depend in particular on the scene and the mesh models used. This is due to the acceleration structures for hierarchical space decomposition, i.e., bounding volume hierarchies (BVHs), applied by OptiX. In our model, we apply treelet restructuring BVH (trBVH) as acceleration structure. RT cores are Nvidia’s special-purpose compute cores for ray tracing and BVH hardware acceleration, which were introduced in the Turing microarchitecture [40]. On Nvidia GPUs without RT cores, software acceleration is used, besides the parallelization on CUDA cores. The ray tracing of the scene with active RT cores is also given in Table 2, showing faster ray tracing execution overall, with a ∼25% increase for doubling the number of rays.

To provide an additional estimate of the timings for a scenario, compute times were taken for the scenario presented in [27] (RTX 2080, RT cores active). The results are shown in Table 2 for the scenario case. The scenario consists of 40 time steps and 40 point clouds are generated. A supplementary animation (in gif-format) of the scenario can be found in the Supplementary Materials. The benefit of the parallelization on the GPU using OptiX affects both ray tracing and object transformations. The time duration includes reading OSI object data updates and copying the generated point clouds from the GPU to the host. However, writing results to a file or a stream on the host are excluded and much more time-consuming.

### 5.2. Validation of Ray Pattern

**Figure 11:**
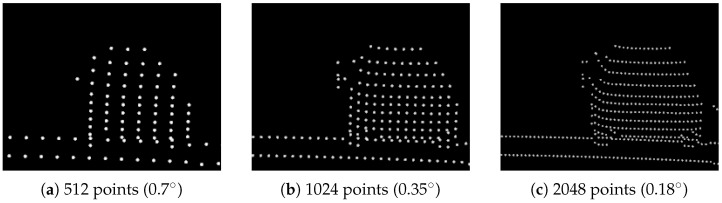
Zooming in on departing car 20 m ahead of ego vehicle on the right lane in Figure 10 using Ouster OS1, 128-layer sensor with different horizontal resolutions (in degrees).

The difference in object resolution becomes apparent when zooming in on the car driving ahead in 20 m distance, shown in Figure 11. Given the object’s width, height and distance from the sensor and the vertical and horizontal resolution, a validation of the ray pattern becomes possible. The departing car in Figure 11 is a 3D model of a Ford Fusion, with 1.72 m width. Thus, with a horizontal resolution of 0.7° (512 pts), seven points are in a vertical line at most. For 0.35° (1024 pts), the number of points doubles to 14 and, as expected, for 0.18° (2048 pts), to 28 points.

The horizontal resolution of a lidar sensor can typically be varied by choosing different update frequencies of the sensor. However, the current version of the model does not cover timing effects such as the update frequency or motion distortion directly. Thus, each time step is a quasi-static scene for which the maximum possible resolution of the ray pattern is assumed.

**Figure 12:**
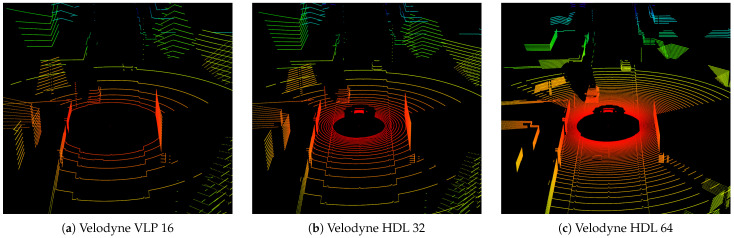
Comparison of point clouds of three lidar sensors from Velodyne: (**a**) VLP with 16 layers, (**b**) HDL with 32 layers and (**c**) HDL 64 layers vertical resolution, all with 0.1° horizontal resolution. A supplementary animation of the scenario with sensor parameterization (**b**) can be found in the Supplementary Materials.

Different vertical resolution is demonstrated in three point clouds of different sensors from Velodyne, shown in Figure 12. VLP with 16 layers, HDL with 32 and HDL with 64 layers are compared. The simulated 360° lidar sensor is located in the center of the concentric circles, on top of the ego vehicle, which is the primary vehicle simulated, at around 2 m height above the street for a representative scene of two cars passing by each other on opposite sides of the street. The street is surrounded by pavement, houses, fences and trees. For the visualization, a rainbow color map was used colored from red to blue with respect to the distance from the sensor origin. All surfaces were modeled with a Lambertian target of 95% from our IR database.

### 5.3. Modular Component

LiMOX is designed as a modular software component in the virtual testing tool chain and therefore has certain limits, which directly impact the physical fidelity of the point cloud. Since the model is not coupled to or integrated into an environment simulation, there are no animations, bump maps or micro-motions available to further improve surface resolution or dynamic motion effects. The point clouds depend highly on the underlying geometry shape. This can be observed in Figure 10 and Figure 12 for the trees, due to their simple geometry. In a rendered image, textures convey a plastic impression. Moreover, geometry meshes sometimes contain support surfaces, which are not part of the actual solid object modeled, like the street markings that are elevated compared to the ground plane; see also Figure 10 and Figure 12.

The primary advantage of this modular ray tracing model is the direct access to the ray generation and processing pipeline, which is often part of the environment simulation itself. In particular, the interfaces and the access to low-level geometry information highly differ from simulation tool to simulation tool. Our approach of a modular ray tracing model, directly based on a ray tracing engine, enables the model developer to handle the ray tracing programmatically and in an independent fashion during model development.

The model facilitates the inclusion of the effects from physical sensors based on ray tracing. We use the model to study different sensor effects, which are observed in lidar data, and try to recreate them to find physical relations.

For the virtual testing of driving functions, object lists are typically used as input. Therefore, an intermediate component is necessary for post-processing raw point cloud data. Such modules include segmentation, clustering and object detection algorithms. For any of these algorithms, the raw point cloud can serve as an input for testing and validation. Moreover, low-level sensor fusion algorithms, based on point cloud data, can be tested using the generated point clouds.

## 6. Conclusions and Discussion

The presented lidar model toolbox is a stand-alone software module, based on ray tracing in OptiX, which operates on 3D geometry models and uses OSI data for scenario generation. It addresses the challenge to perform ray tracing outside of an environment simulator, as an independent software module.

To simulate the lidar principle, surface materials and the incidence angle are considered for the ray–surface interaction. Two different IR material databases can be linked to the sensor model and a material mapping needs to be provided for all materials defined in the geometries. Material classes are introduced for handling different kinds of materials with respect to their transparent, absorbent, reflective and retroreflective attributes, which invoke different hit programs. They can be easily extended to simulate new observed effects.

Moreover, from the reflectivity of the material, a maximum possible range is modeled with respect to the data sheet parameters provided. Different functional relations are compared. The IR materials used together with the range–reflectivity relations modeled have a high impact on the point clouds generated, as shown.

The applications of the model are to generate point clouds for a single scene or an entire scenario. The simulated point clouds are useful for the virtual testing and development of perception or sensor fusion algorithms. The model is highly parameterizable to simulate various sensors and environment conditions. It is meant to be used and extended with measurement data and is easily adaptable for different functional relations. Various sensor ray patterns can be simulated, including predefined existing sensors or user-defined sensors. Spatial resolution is studied for different sensor types and ray patterns and validated for a vehicle of known size.

The quality and realism of the point cloud are highly dependent on the 3D models used, their surface properties, surface resolution and material association. For a comparison with sensor measurements, a digital twin of the environment, the scenario and the dynamic objects is needed.

Moreover, the model does not reproduce the time resolution of a physical sensor and its update frequency, but only updates the point cloud on changes in the scene. A time resolution of the shooting pattern is currently not included and dynamic effects, such as distortion, are neglected. Due to the high efficiency of ray tracing using the OptiX engine on the GPU, this is not a firm limit of the model, and these effects could be included in the future. An OptiX method exists to simulate motion blur [41], which interpolates two given points of an object’s trajectory in space, yielding a couple of intermediate points on a linear path in between, where polygons are shifted to, for creating motion blur hit points. This effect seems suitable to simulate the motion distortion effects of point clouds for small, linear displacements.

To deploy the model in a co-simulation environment with high demands on efficiency or even real-time performance, OptiX was chosen for parallelization on the GPU. A rough estimation of the compute times for a scene and a simple scenario is presented and compared for doubling the number of rays, respectively. This demonstrates the GPU acceleration benefit for large numbers of rays.

Well-known sensor effects like beam divergence are currently not considered in the model. Therefore, multi-echo detections from a single pulse or ray, hitting multiple surface targets, cannot be simulated at this point. This effect is often caused by vegetation, due to the multiple discrete surfaces hit by a larger beam cross-section. The meshes used for vegetation are mostly enhanced by textures and, thus, the polygon geometry models do not reflect the detailed physical shapes, which is another challenge for point cloud generation.

Our model was developed to simulate pulsed (ToF) lidar sensors, which are currently more common in automotive applications. Continuous-wave sensors show further sensor effects and need to be analyzed separately.

## 7. Outlook

The mapping of the IR materials to the geometry models is still an open topic, which ideally is resolved during mesh generation. The current option is to assign each visual material an IR material from the database. This is, however, not sufficient, since visual material definitions often do not reflect the physical properties needed. Therefore, a standardization of the mesh models used together with material association at the polygon level is an ideal solution to this challenge and has been proposed in the glTF format as a standard by the Khronos Group [42] and its extension to OpenMaterial [43] definitions.

Within glTF, all layers of graphics descriptions are present and can be addressed during a data exchange or association. This includes scene, node, mesh, view point, materials, animation, texture or images, which are covered and can be addressed in a general fashion. OpenMaterial is a proposed extension to address broader material properties across the electromagnetic and acoustic spectrum, as well as mechanical, thermal or surface attributes, e.g., surface roughness or surface coatings. These additional properties of surface and material characteristics could directly be used for better mapping opportunities of the physical surface and the IR database integration, for geometry models using OpenMaterial definitions.

The validation with respect to the material dependence is a work in progress. For this, we will use a 3D object reconstruction from high-resolution lidar data to generate a static scene from a testing ground and compare it to a point cloud from a typical automotive lidar sensor, such as Ouster OS1. Prerequisites for the validation are the correct association of materials to the 3D models and the appropriate choice of material measurements from the IR database. This material mapping process is a research topic on its own and needs to be conducted separately.

**Figure 13:**
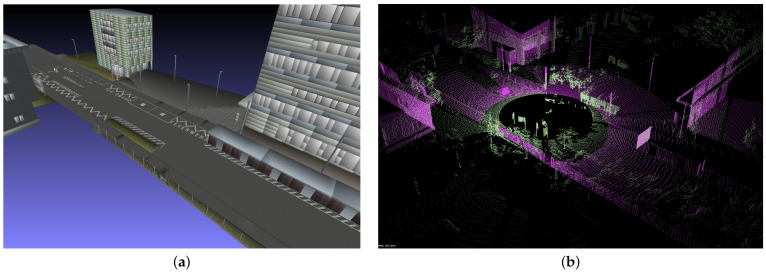
Comparison of model data and measurements: (**a**) 3D geometry model. (**b**) Model point cloud (pink) compared to measurement data (green) of an Ouster OS1 sensor of 128 layers. In this example, the sensor origin is not co-aligned.

A first attempt to model an existing 3D environment and compare the resulting point cloud to measurement data is shown in Figure 13. However, all surfaces are still modeled with the same reflectivity of 50%. Many objects are present in the measurement which are not covered by the 3D model environment. These are dynamic objects, temporary objects, vegetation (hard to model in detail) as well as seasonal effects or changes due to environment conditions (e.g., wind or snow). Moreover, additional sensor effects, such as sensor noise or signal processing details, need to be taken into account in the comparison or added to the simulation.

Our model can be extended to simulate multi-path reflections by launching new rays off from mirror or semi-transparent surfaces. Noise and uncertainty models for more realistic point clouds are currently not included. For both effects, data analysis and probability estimation are needed to quantify their occurrence.

Another topic of interest is the data exchange of the model with the environment simulation. A closer interoperation with different simulation tools can improve the point cloud fidelity, providing, for example, mesh refinement, bump or displacement maps, micro-motion or animation. For this, standardized interfaces are needed, such as glTF [42] or the External Sensor Interface (ESI) [44] as proposed by CARLA [9] developer Daniel Santos-Oliván. He proposes a common interface for sensor simulation of different environment perception sensors, like radar, lidar or camera. The proposed interface is independent from any environment simulation and includes a physics engine, a ray tracing engine and a core with access to an open scene graph on which the 3D scene is based and the models can operate together with a sensor manager and I/O update. The user can access it manually, access it or via a bridge by CARLA.

Even though the interfaces to fully connect the model modularly with different environment simulations are missing, the presented modular lidar model based on ray tracing is an alternative to creating the point cloud in an environment simulator directly. The model is limited by the lack of available and standardized geometry models including physical material property associations. We think that the development and application of a modular architecture should be encouraged in the simulation community for realistic simulation models.

## Figures and Tables

**Table 1:** Overview of different sources for parametrization.

Data Sheet	Data Analysis,	Scenario,
	Measurements	Sim. Context,
		Sim. Environment
sensor parameters:	range–reflectivity	object data:
FOV, resolution,	dependence	3D geometry model,
ray pattern,		ID, pose, update
range limits (min, max)		
range–reflectivity pairs:	attenuation	material–surface
$\left[r_{max},R\%\right]$	measure	association
		weather condition
		time step

**Table 2:** Time profiling: ray tracing the scene, presented in Figure 10, with and without active RT cores, and a scenario of 40 time steps presented in [27] with active RT cores, including object updates.

Ouster OS1, 128	Duration (ms)		
hrz. res.	512 pts	1024 pts	2048 pts
case	(0.7°)	(0.35°)	(0.18°)
single scene	1 ms	1.16 ms	1.35 ms
(RT cores inactive)			
single scene	0.65 ms	0.8 ms	1 ms
(RT cores active)			
scenario (40 scenes)	37 ms	51 ms	74 ms
(RT cores active)			

## Data Availability

Data is contained within the article and Supplementary Materials.

## Supplementary Materials

The following supporting information can be downloaded at: https://www.mdpi.com/article/10.3390/s24061846/s1, Two animations of the point clouds for a simulated scenario can be found online: Animation of Figure 10b: Fig10b_Ouster128_1024_t95_fv.gif, animation of Figure 12b: Fig12b_VelodyneHLD32_t95_fv.gif.

## Nomenclature

A summary of all symbols and significant abbreviations can be found below:

**Symbols**	
*r*	range, distance (m)
$r_{L}$	range limit (m)
*R*	reflectivity (%)
$R_{ref}$	reference reflectivity value (%)
ρ	reflectance (%)
λ	wavelength (nm)
θ	incidence angle (°)
p	point location
n	face normal of hit polygon
**Abbreviations**	
AH	any hit
API	application programming interface
BRDF	bidirectional distribution function
BVH	bounding volume hierarchies
CH	closest hit
CPU	Central Processing Unit
CUDA	Nvidia’s API for GPU programming
DB	database
FMU	Functional Mock-up Unit
FOV	field of view
GPU	Graphics Processing Unit
I/O	input/output
IR	infrared
OS1	Ouster sensor (version 1)
OSI	Open Simulation Interface
PBR	physically based rendering
PCD	point cloud data (data format)
RT	ray tracing
SDK	software development kit
TCP/IP	Transmission Control Protocol/Internet Protocol
ToF	time of flight
VTD	Virtual Test Drive (simulator)
