# Supplementary figures and images for: LiMOX—A Point Cloud Lidar Model Toolbox Based on NVIDIA OptiX Ray Tracing Engine
Source: Sensors (Basel). 2024 Mar 13;24(6):1846. doi: 10.3390/s24061846 (PMC10975039; doi:10.3390/s24061846)

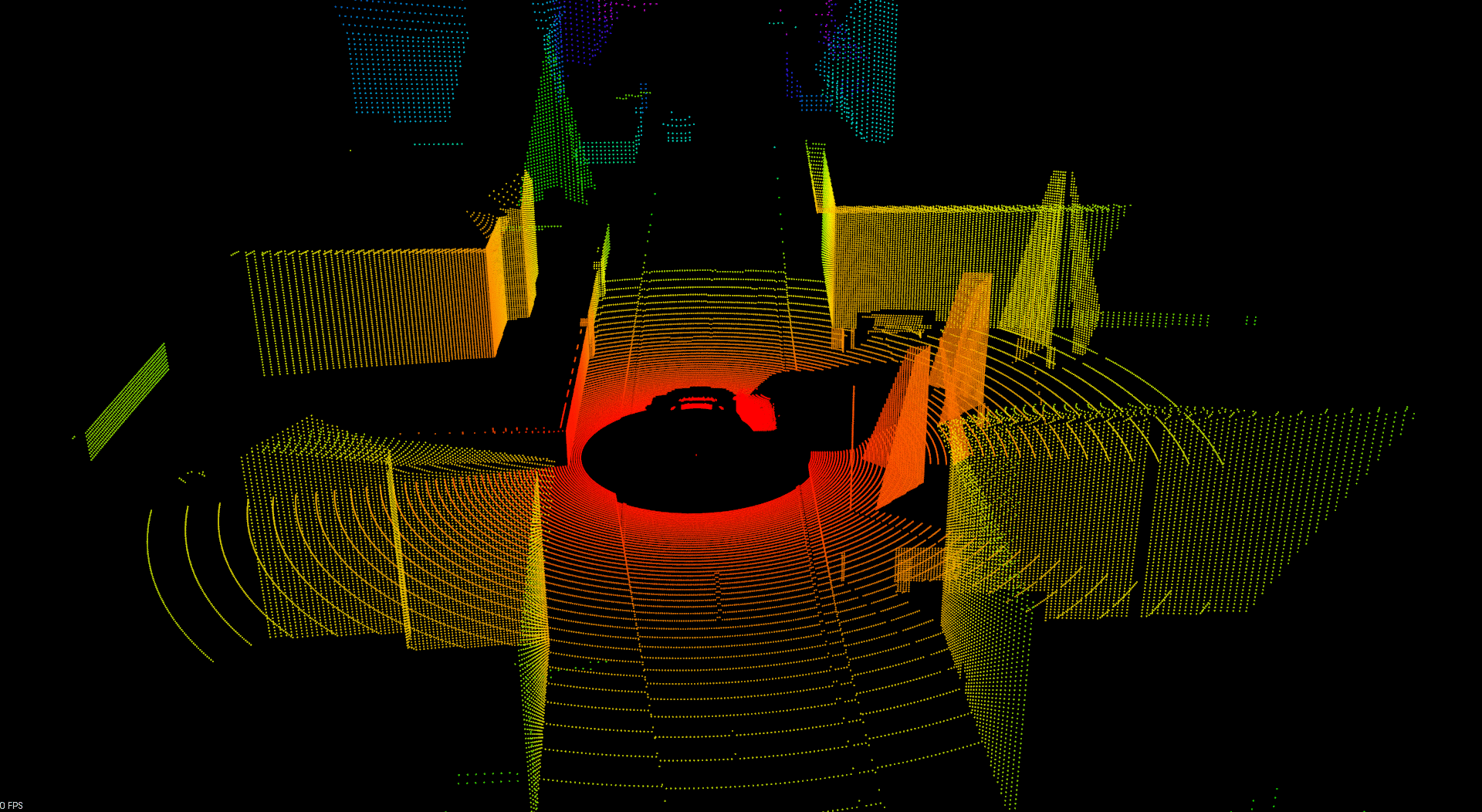

Supplement: Supplementary file 1 [file sensors-24-01846-s001.zip › Fig10b_Ouster128_1024_t95_fv.gif]

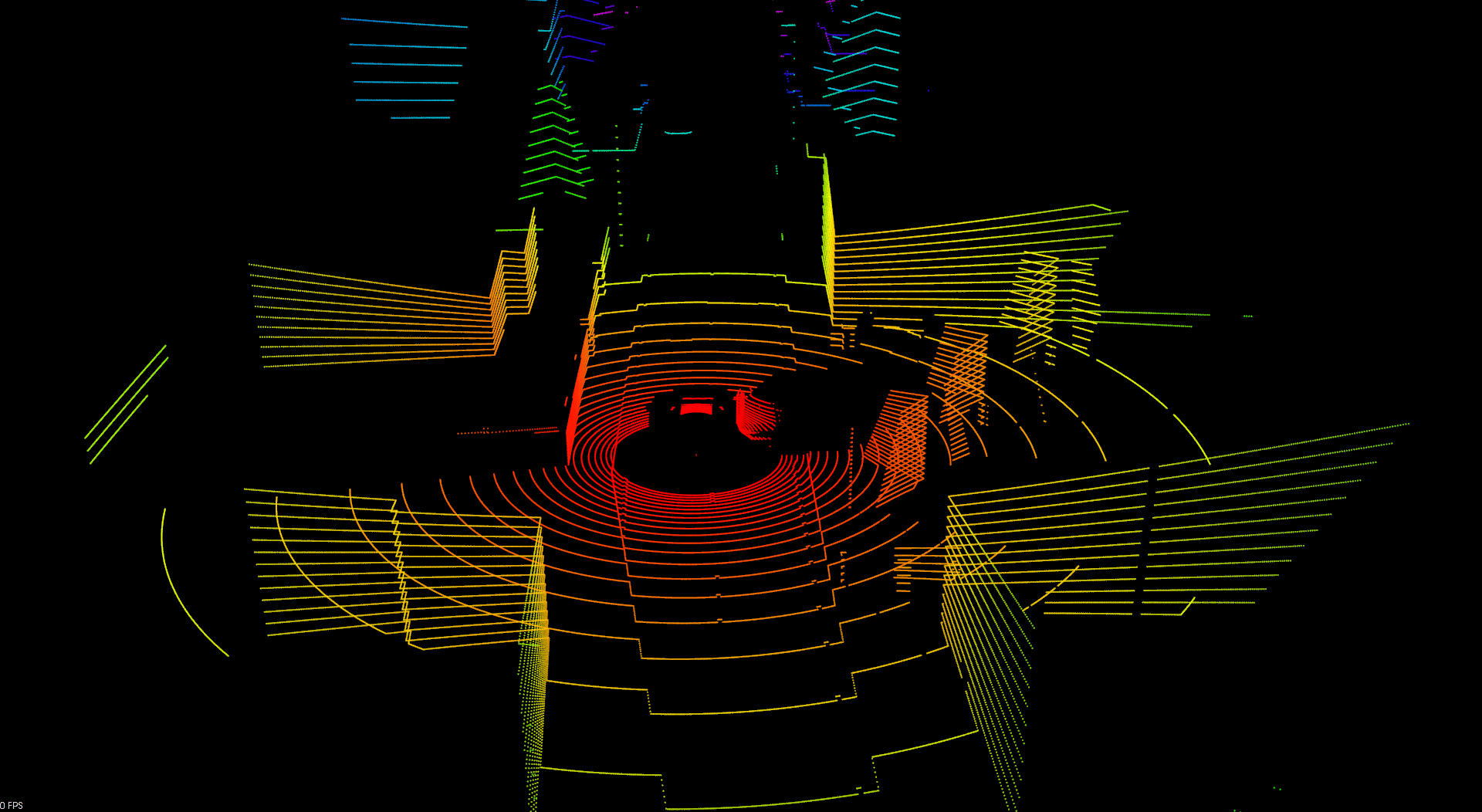

Supplement: Supplementary file 1 [file sensors-24-01846-s001.zip › Fig12b_VelodyneHLD32_t95_fv.gif]
